# Increased pacemaker implantation and mortality rates in relatives of patients with early-onset sinus node dysfunction: can genetics explain all?

**DOI:** 10.1093/europace/euae289

**Published:** 2024-11-13

**Authors:** Giulio Conte

**Affiliations:** Division of Cardiology, Cardiocentro Ticino Institute, Ente Ospedaliero Cantonale, 6900 Lugano, Switzerland; Faculty of Biomedical Sciences, Università della Svizzera Italiana (USI), 6900 Lugano, Switzerland

## Abstract

**Graphical Abstract:**
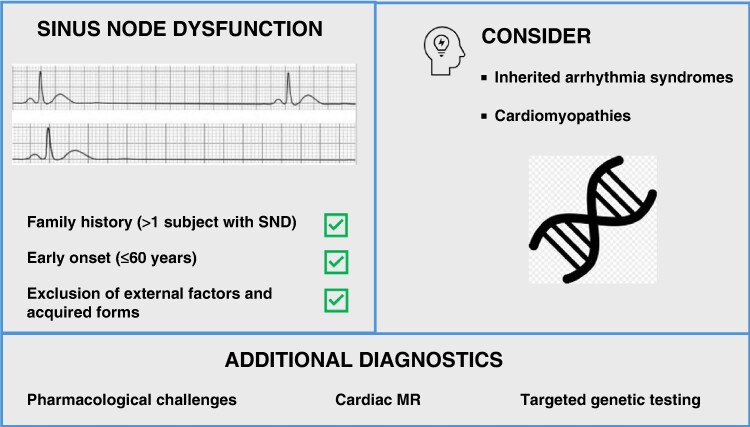

**This editorial refers to ‘Familial risk of sinus node dysfunction indicating pacemaker implantation: a nation-wide cohort study’ by M.K. Christiansen *et al.*, https://doi.org/10.1093/europace/euae287.**

Sinus node dysfunction (SND) is a cardiac conduction disorder with an incidence that increases with age, and it is commonly related to age-dependent sinoatrial node structural and electrical abnormalities or to external factors and acquired forms, including systemic and cardiovascular diseases.^1^ When SND appears earlier in life and extrinsic factors are not present, it may indicate the presence of different underlying causative mechanisms.

In this issue, Christiansen *et al.*^2^ report on the impact of family history of SND requiring cardiac pacing and the relationship between temporal onset of SND in the index case and the risk of SND and mortality in first-degree relatives.

In the retrospective population-based study, the rate of pacemaker (PM) implantation for SND in the general population over a 40-year period (1982–2022) was retrieved from three national Danish registries and compared to the rate of PM implantation in first-degree relatives of patients with a PM due to SND. The cumulative incidence of PM implantation for SND in the general population was 0.03% and 0.27% at 50 and 68 years. Notably, subjects having a first-degree family member with a PM due to SND had a 2.9-fold increased risk of developing SND requiring a PM, with a cumulative incidence of SND of 0.10% and 0.79% at 50 and 68 years, respectively. Moreover, the risk of PM implantation due to SND was inversely associated to the age of PM implantation in the index patient with a 5.5-fold increased risk for family members of patients receiving a PM before the age of 60 years (cumulative incidence of 0.19% and 1.45% at 50 and 68 years).

These findings, despite the absence of genetic data in the studied population, may suggest the presence of a substantial genetic contribution to SND in families with multiple family members carrying a PM because of SND. It has been already reported that early-onset cardiac conduction diseases of unknown origin can have a genetic basis and different genes encoding ion channels (*HCN4*, *SCN5A*, *GNB2*), sarcomere proteins (*MYH6*) and lamin A/C (*LMNA*), or intermediate filaments (*KRT8*) are implicated in SND.^3–8^ A genome-wide association study combined with polygenic score analysis and a Mendelian randomization found an association between six loci and SND.^8^ Apart from a novel low-frequency missense variant within the *KRT8* gene, encoding keratin 8, two variants within cardiac structural proteins (*MYH6* and *TTN/CCDC141*), one within a sodium channel (*SCN10A*), there were two variants previously described as atrial fibrillation (AF) risk loci encoding transcription factors (*ZFHX3* and *PITX2*).^8^ It is well recognized that SND is often associated with AF and it is not surprising that in this study, up to 47% of index patients with SND and PM presented with AF.

Based on the evidence of potential multiple gene-determined pathways implicated in SND and the relative low diagnostic yield of genetic testing in SND patients (<25%), the 2022 EHRA/HRS/APHRS/LAHRS expert consensus statement on genetic testing states that genetic testing ‘may be considered’ in unexplained SND cases, especially in the presence of a positive family history, with a targeted panel that should include the core genes *SCN5A*, *HCN4*, and *LMNA*.^9^ Although there is no specific recommendation for genotype-based risk stratification for SND patients, a positive genetic testing may have prognostic implications as many genes associated with SND are also implicated in different forms of genetic cardiac diseases.

It is well established that SND can be one of the phenotypic features of different inherited arrhythmia syndromes including Brugada syndrome (BrS), catecholaminergic polymorphic ventricular tachycardia (CPVT), long QT syndrome (LQTS), and progressive cardiac conduction disease (PCCD).^9–12^ A proper diagnostic assessment directed to inherited arrhythmia syndromes and cardiomyopathies in these patients may include pharmacological challenges, cardiac imaging studies (e.g. cardiac magnetic resonance), and targeted genetic testing. A specific diagnosis of a cardiac inherited electrical or structural disease carries also important implications in terms of device selection, ventricular arrhythmias risk stratification, tailored pharmacological treatment, and family screening strategies. Sinus node dysfunction has a prognostic value in BrS, especially in the paediatric population and is related with ajmaline-induced life-threatening arrhythmias.^13,14^ In BrS patients undergoing continuous monitoring by implantable loop recorders, SND is present in up to 8% of adults and is the cause of syncope in 64% of cases.^15^ Unexpectedly, despite the younger age, a similar prevalence of SND (9%) is present in the paediatric BrS population.^16^ Additionally, in a large cohort of patients with atrial arrhythmias and channelopathies, SND was found in 10% of cases, ranging from 10% in patients with BrS and LQTS to 20% in patients with CPVT and PCCD.^17^ Therefore, before genetic testing, a pharmacological challenge with sodium channel blocker should be considered in patients with early-onset SND and suspicion of BrS.

Interestingly, in this study, the mortality rate was significantly increased in individuals with a family history of early-onset SND with a 1.22-fold increased risk among individuals having a mother, father, or sibling implanted with a PM for SND. As stated by the authors, due to the nature of the study, the specific causes of death could not be ascertained. However, there was an adjusted 1.2-fold increased risk of cardiac arrest and ventricular arrhythmias in family members of an index patient with SND and a PM implanted before the age of 60 years. In this study, it was not determined if these patients were carriers of a pathogenic gene variant related to channelopathies or cardiomyopathies at risk of ventricular arrhythmias. Therefore, the observed correlation between early-onset SND and the risk of sudden death and ventricular arrhythmias in the relatives remains to be assessed.
